# Prevalence of Bilateral Asymmetry of Tibial Bones Length in MBBS Students of A Medical College

**DOI:** 10.31729/jnma.4548

**Published:** 2019-08-31

**Authors:** Iju Shrestha, Bansi Krishna Malla

**Affiliations:** 1 Department of Anatomy, Kathmandu Medical College and Teaching Hospital, Duwakot, Bhaktapur, Nepal

**Keywords:** *bilateral asymmetry*, *tibial length*

## Abstract

**Introduction:**

A wide variability of bilateral asymmetry in human has been observed within the population. However sufficient attention has not been given to the difference present in the limbs especially the tibial bones. It is generally assumed that the both limbs of the individual are with insignificant differences. The objective of the study is to find the prevalence of bilateral asymmetry of the tibial bone length of the same individual and distribution in between the two genders.

**Methods:**

This descriptive cross-sectional study was conducted on 150 students of Kathmandu Medical College and Teaching Hospital after obtaining ethical approval. Simple Random Sampling technique was used. The right and the left tibial length were recorded for different genders

**Results:**

Bilateral asymmetry in the tibial bone length was observed in 66 (44%) [41.58%- 46.42% at 95% CI] of the subjects which was recorded more in males 98 (65.15%) than in females. The minimum and maximum differences between the tibial length present was 0.1mm and 0.8 mm respectively with a mean of 0.2136 mm. Among the three age groups, tibial length asymmetry was observed highest 67 (45.56%) in Group B (20-22 years). Asymmetry in length was seen more in the right tibia with male preponderance over female.

**Conclusions:**

Asymmetry in the tibial bones length should be given proper attention and proper diagnosis and treatment of leg discrepancies should be done.

## INTRODUCTION

Species wide bilateral asymmetry in human has been observed and has been reported with wide variation in morphologic features of lower extremity within and across populations.^1,2^ Despite this, limb asymmetry within individuals is often overlooked.^2^ It is generally assumed that the left and the right extremities are not significantly different, because of which contralateral bones are often used as a reference in clinical, forensic and anthropological studies.^3^ At first glance, this deformity may seem fairly simple to diagnose and treat.

However, proper investigation into the etiology and the associated compensatory mechanisms should be done. Moreover, there have been very few studies regarding the length of tibial bones. Keeping this in view, various studies have been done about the leg length discrepancies in which whole length of the limb is taken. But there has been very less study about the variation in the tibial bone length of the same individual.

The aim of this study is to find out the prevalence of asymmetry in tibial bones length of same individual and magnitude of the same in males and females.

## METHODS

This descriptive cross-sectional study was carried out in Kathmandu Medical College and Teaching Hospital from January 2019 to April 2019. Ethical clearance from Institutional Review Committee, Kathmandu Medical College and Teaching Hospital- Ref No.-2812201803, was obtained. The study enrolled 150 students of seventeen to twenty-five years of age. The sample size was calculated with prevalence 50%.

Sample size (n) was calculated as:

n= Z^2^P (1-P)/e^2^

  = (1.96)^2^ X 0.5 (1-0.5)/ (0.08)^2^

   = 150

where

P=0.5,
z=1.96 for the confidence interval is 95%,
e=0.08

Simple random sampling technique was used. Informed consent of participants was taken prior to the procedure. Age, sex and length of tibia of both legs of the participants were recorded. As the study deals with the length of tibia, history of any disease/ deformity/ injury/fracture or surgical procedures of the leg was also taken.

For obtaining the tibial length, the subject was asked to sit straight with thigh in a straight line, knee flexed at 90° position and the foot was rotated laterally, which makes the bony projections prominent. Then, length of the tibia was measured as a straight distance between the superior-most margin of the medial condyle to the inferior-most margin of the medial malleolus.

The data obtained was computed and analyzed using Excel to tabulate the results of mean, median, mode and frequency.

## RESULTS

**Figure 1.**
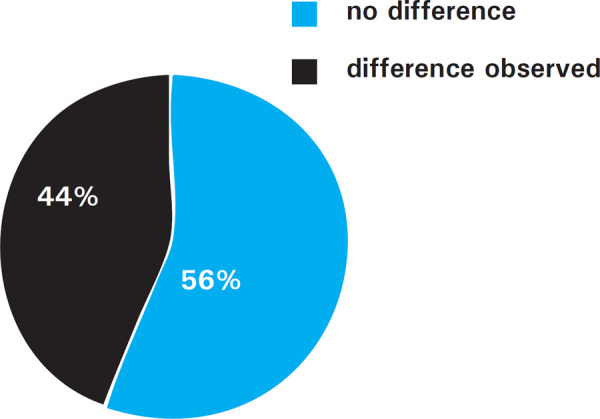
Presence of bilateral asymmetry of tibial bones length.

In this study, 150 participants included 82 males and 68 females. Bilateral asymmetry of the tibial bones length was observed in 66 out of total participants (Figure 1).

**Figure 2.**
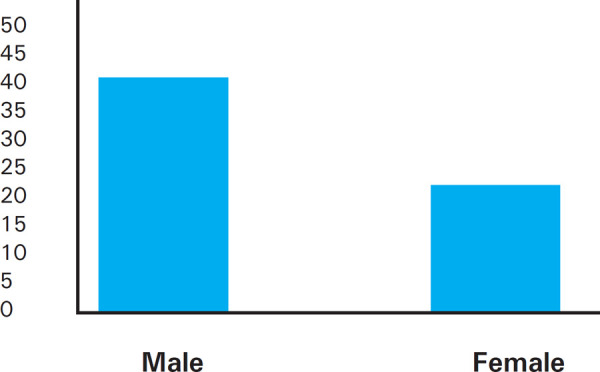
Sexual dimorphism in bilateral asymmetry of tibial bones.

Among the participants, asymmetry of tibial bones was seen more in males than in females (Figure 2).

The minimum difference between the tibial bones present was 0.1mm while the maximum difference of 0.8 mm with a mean of 0.2136 mm. The mode and median was each observed to be 0.2.

**Figure 3.**
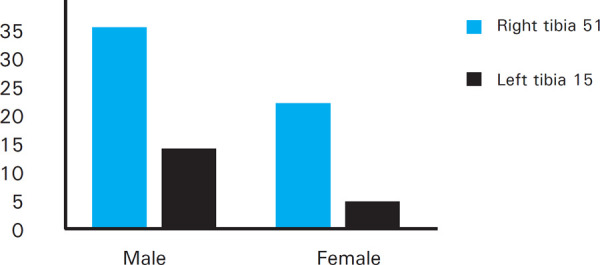
Asymmetry difference observed in the tibial bones dimension.

Among the asymmetries, the difference in dimension was exhibited more by the right tibia 51 (77.27%) than the left 15 (22.7%). (Figure 3)

**Figure 4.**
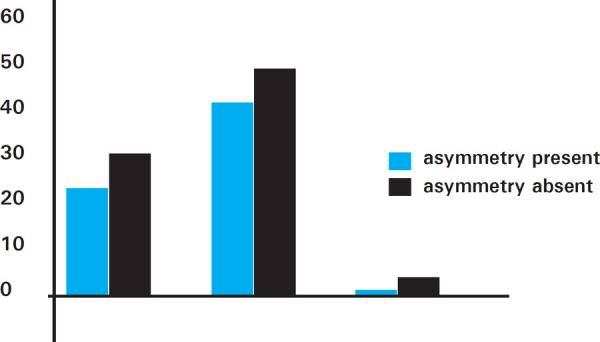
Bilateral asymmetry of tibial bones observed in different age groups.

The subjects’ age was divided into three groups of A: 17-19 years, B: 20-22 years, C: 23-25 years. Among the age groups, the subjects were more in Group B (90). Group A included 55 subjects whereas the least were listed in Group C (5). Bilateral asymmetry was exhibited by 43.63% in Group A while 45.56% and 20% in Groups B and C respectively (Figure 4).

**Figure 5.**
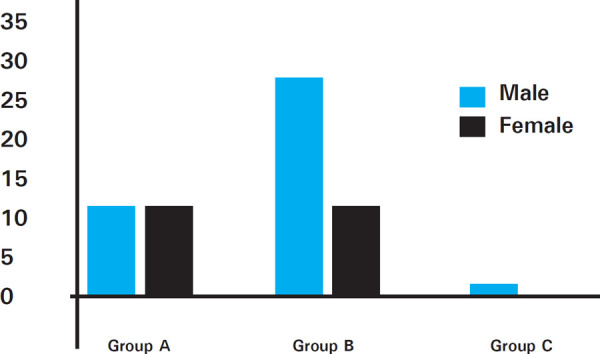
Sexual dimorphism in various age groups exhibiting bilateral asymmetry.

Males dominated in Group B while the sexual dimorphism was equal in Group A. The only subject exhibiting bilateral asymmetry was male in Group C (Figure 5).

## DISCUSSION

Asymmetries in the right and the left extremities of an individual are generally assumed to be insignificant. Due to this assumption contralateral bones are used to serve the purpose of reference in various studies. Also discrepancy in length between the extremities has been reported to be frequent in the population between 40 and 70% being affected.^4^ Among the results of asymmetry, McCaw describes stress fractures, back pain and knee joint pain.^5^ Other studies have shown leg length discrepancy to be frequently associated with hip pain, sciatica, muscle fatigue.^6^ The data in our study shows the presence of asymmetry (44%) between the right and the left tibial bone lengths. With difference range of 0.1mm-0.8 mm with a mean of 0.2136 mm, the data may be considered to affect the length of the leg as a whole. The study is consistent with the studies done by Auerbach and Ruff and Plochocki where bilateral asymmetries in the long bones have been reported in terms of different elements including the tibial length.^1,7^ Our finding also coincides with the findings of K. Krishan et al where significant asymmetry has been reported in the lower leg length.^8^ In contrast, Radzi et al, who have used the 3D bony models reconstruction from CT scan, have reported insignificant differences in the tibial dimensions.^3^ Sexual dimorphism in this study showed preponderance of the males (65.15%) over female. This result is in contrast with the findings of Auerbach and Ruff where they reported least asymmetry in the sexes in terms of tibial length.^1^ However in their finding, asymmetry in tibial length in males still showed significant correlation over females.

Asymmetry observed in the tibial bone length was more in the right tibia (77.27%) in our study. Studies related to behavioural preferences for the lower limb laterality have also shown higher frequencies of right footedness^9,10^ Foot preference in the behavioral studies was assessed as the foot utilized for object manipulation or other activities involving motor coordination -most commonly kicking, but also picking up objects, tracing or drawing with the foot, tapping, or stamping. Similar studies concerning the leg length differences have also mentioned that the left leg is generally the shorter one when the leg difference is present.^11^ The findings of these studies together with our study may be explained in terms of lateralized limb preference. The comparatively longer length of the right tibia may be due to the active right limb preference. However, Auercbach and Ruff have reported more asymmetric left tibia than the right in Black Americans.^1^ Asymmetrical length in the female, in this study, also showed longer length of right tibia which was in contrast to the study by Ruff and Jones where left tibial dimensions of females were more.^12^

Among the three age groups we had in our study, asymmetrical tibial length was observed more (45.56%) in group B which had the participants age ranging from 20-22 years. In group A the asymmetrical tibial length was a little less than group B whereas that was the least in Group C which included age 22-25. Very little has been explained about the tibial length in terms of age groups. Radzi et al has mentioned the criteria as their limitation of the study.^3^ However, studies under various age groups regarding laterality preference (of foot) have been done. Gabbard and Iteya found there was a prominent shift to right sidedness during late childhood, after which the behavior remains almost stable with smaller difference in the adolescent and adult.^10^ This finding is comparable to our study where we also observed asymmetry present more in the middle group (B) than in other groups and it declined in group C. this finding may be attributed to behavioural and developmental changes which needs further detailed consideration.

A limitation of this study is small differences reported between left and right tibia which may have minimal clinical relevance. Nevertheless with the prevalence of 44% in the tibial bone alone in this study definitely shows the need of further detailed study regarding the bilateral asymmetry of tibial bones dimension that will ultimately reflect asymmetry in leg length as a whole. Secondly, the range of the age of the subjects could have been extended so that further detailed study of the asymmetry of limbs in much younger and older age groups would have been possible.

## CONCLUSIONS

Owing to smaller differences (mean of 0.21mm) in the right and left tibial length of the same individual, the contralateral tibia may be taken into consideration for different study purposes such as clinical, forensic or anthropological. However prevalence of the bilateral asymmetry of tibial bones length observed in this study (44%) cannot be neglected. This discrepancy will finally be reflected in the leg length which can be the underlying cause of different disorders such as backpain, osteoarthritis etc. Hence knowledge of these asymmetries and proper investigation put together can result in a proper diagnosis and treatment.
